# Longitudinal Evaluation of Segmental Arterial Mediolysis in Splanchnic Arteries: Case Series and Systematic Review

**DOI:** 10.1371/journal.pone.0161182

**Published:** 2016-08-11

**Authors:** Hyun Soo Kim, Sang-il Min, Ahram Han, Chanjoong Choi, Seung-Kee Min, Jongwon Ha

**Affiliations:** Department of Surgery, Seoul National University College of Medicine, Seoul, Korea; China Medical University, TAIWAN

## Abstract

**Background:**

Segmental arterial mediolysis (SAM) is a rare non-atherosclerotic, non-inflammatory vascular disorder varying widely in clinical course. The purpose of this study is to analyze detailing clinical and imaging manifestations over time in patients with SAM through a literature review and to suggest an optimal management strategy.

**Methods:**

A retrospective review of eight consecutive patients diagnosed with SAM between January, 2000 and January, 2012 was conducted. All presented with acute-onset abdominal or flank pain. Clinical features, imaging studies, and laboratory findings served as grounds for diagnosis, having excluded more common conditions (ie, fibromuscular dysplasia, collagen vascular disorders, or arteritis). CT angiography was done initially and repeated periodically (Week 1, Month 3, then yearly). Treatment was conservative, utilizing endovascular intervention as warranted by CT diagnostics. In a related systematic review, all English literature from 1976 to 2015 was screened via the PubMed database, assessing patient demographics, affected arteries, clinical presentations, and treatment methods.

**Findings:**

Ultimately, 25 arterial lesions identified in eight patients (median age, 62.8 years; range, 40–84 years) were monitored for a median period of 26 months (range, 15–57 months). At baseline, celiac axis (3/8, 37.5%), superior mesenteric (4/8, 50%), and common hepatic (2/8, 25%) arteries were involved, in addition to isolated lesions of right renal, splenic, right colic, middle colic, gastroduodenal, left gastric, right gastroepiploic, proper hepatic, right hepatic, and left hepatic arteries. Compared with prior publications, celiac axis and superior mesenteric artery were more commonly affected in cohort. Arterial dissections (n = 8), aneurysms (n = 5), stenoses or occlusions (n = 4), and a single pseudoaneurysm were documented. Despite careful conservative management, new splanchnic arterial lesions (n = 4) arose during follow-up. Considering the few available reports of new arterial lesions in the literature, newly developing pathology is a distinctive feature of our patients, four of whom eventually required endovascular interventions.

**Conclusions:**

Careful clinical observation via periodic CT angiography is required in patients with SAM, checking for newly developing lesions. The natural history of SAM should be clarified in a larger patient population.

## Introduction

Segmental arterial mediolysis (SAM) is a rare non-atherosclerotic, non-inflammatory vascular disorder that varies considerably in clinical course. Its`etiology remains unclear, although some consider it a variant or a precursor of fibromuscular dysplasia (FMD) [1, 2]. Others have suggested that SAM is a secondary phenomenon, resulting from vasospasm or arterial wall injury due to immune complexes [3]. Originally described in 1976 by Slavin et al [4], only around 100 cases of SAM have since been reported in the literature [5].

SAM is typically manifested in splanchnic arteries where lytic degeneration of medial smooth muscle occurs, culminating in tearing/separation from adventitia and adjacent fibrosis. Patients variably present with dissection, aneurysm, stenosis, occlusion, or hemorrhage after rupture, often calling for emergency surgical or endovascular intervention. Unfortunately, no standard criteria exist at present for differentiating SAM from inflammatory vasculitis, and the clinical course of SAM is varie, with no clear tendency to progress, resolve, or stabilize. Likewise, there are no established therapeutic or monitoring guidelines stipulating circumstances where surgical or other interventions are indicated.

The purpose of this study is to review our experience with SAM management and to suggest an optimal management and surveillance strategy in SAM through thorough literature review.

## Methods

A retrospective review of eight consecutive patients (male, 4; female, 4) diagnosed with SAM between January, 2000 and January, 2012 was conducted with approval of the Institutional Review Board (IRB No SNUH-1408-027-601). The obtainment of informed consent was waived and patient information/records were anonymized and de-identified prior to analysis.

### Diagnosis of SAM

Diagnostic criteria developed by Kalva et al [6] were applied, relying upon presenting clinical features, imaging studies, and laboratory findings. Briefly, patients experiencing acute or chronic abdominal pain, flank pain, or no symptoms qualified as SAM, in the absence of any congenital predisposition for dissection (eg, Ehlers-Danlos, Marfan’s, or Loeys-Dietz syndrome) and after excluding more common alternatives, namely FMD, collagen vascular disorders, or arteritis. On imaging studies, dissection, aneurysm, or occlusion involving multiple splanchnic arteries was characteristically found findings. No inflammatory markers of vasculitis (ie, ANCA, C3/C4, FANA, RF, anti-La antibodies, and anti-cardiolipin antibodies) were elevated on laboratory testing.

### Treatment and Surveillance of SAM

Treatment of SAM generally was conservative, including strict control of blood pressure, resting of the bowel (if mesenteric arteries involved), and close observation, prohibiting use of anticoagulants or antiplatelet agents. CT angiographic studies were repeated at Week 1 and at Month 3 after diagnosis and thereafter were done yearly. On occasion, CT angiography was ordered more often (at physician’s discretion) if new lesions or symptoms developed. In patients requiring superior mesenteric arterial stenting, self-expandable stents were placed in a manner reported previously [7].

### Literature review

PubMed databases were searched for all articles published in English between January 1, 1976 and August 31, 2015. “Segmental arterial mediolysis” and “segmental mediolytic arteritis” served as keyword combinations. All abstracts, case reports, patient series and citations scanned were examined. Extraction of study data was achived using a standardized template, which included author`s name, publication year, patient demographics, affected arteries, clinical presentations, and treatment methods. A full description of search terms, strategy and screening stages are provided in S1 Table.

This literature review follows the Preferred Reporting Items for Systematic Reviews and Meta-Analyses (S1 PRISMA Checklist, S1 PRISMA Flow Diagram).

### Statistical analysis

We analyzed variables of our case series and of those in the literature. Because events were relatively few and biased, the binomial Clopper Pearson exact method was used to generate 95% confidence intervals.

## Results

### Patients demographics

From January 1, 2000 to January 31, 2012, a total of 8 patients were diagnosed as SAM (Table 1). Median age was 62.8 years (range, 40–84 years, 95% CI range, 51.7–73.8 years), and median follow-up period was 26 months (range, 15–57 months). All patients presented with acute abdominal or flank pain. Relevant patient comorbidities included hypertension (25%), atrial fibrillation (25%), congestive heart failure (12.5%), and hyperlipidemia (25%). Only one patient was a current smoker (20 pack-years), the rest denying such habit. None of the patients had family histories or clinical manifestations suggesting congenital predispositions for dissection (eg, Marfan’s syndrome, Ehlers-Danlos syndrome, and FMD).

**Table 1:** Baseline demographic and clinical characteristics of patients.

Variables	Patients (N = 8)	95% CI
Age: median (range), y	62.8 (40–84)	62.8 (51.7–73.8)
Gender (male:female)	4:4	50.0 (15.7–84.3)
Initial presentation as abdominal pain, n (%)	7 (87.5%)	87.5 (47.4–99.7)
Initial presentation as flank pain, n(%)	1 (12.5%)	12.5 (0.3–52.7)
Comorbidities, hypertension, n (%)	2 (25%)	25 (3.2–65.1)
Comorbidities, congestive heart failure, n (%)	1 (12.5%)	12.5 (0.3–52.7)
Comorbidities, hyperlipidemia, n (%)	2 (25%)	25 (3.2–65.1)
Comorbidities, smoking, n (%)	1 (12.5%)	12.5 (0.3–52.7)
Family history of arterial disease	0 (0%)	0 (0–36.9)
Histologic diagnosis	0 (0%)	0 (0–36.9)

Detailed clinical and imaging findings at presentation and during follow-up of patients are summarized in Table 2.

**Table 2:** Clinical and imaging findings at presentation and follow-up.

No.	Age (y) /Gender	Clinical presentation	Follow up (months)	Comorbidity	Initial CT angiography	Newly developed arterial lesion at follow-up CT angiography	Endovascular treatment
1	63/M	Abdominal pain	17	None	SMA dissection	• Progression of SMA dissection • SMA branch aneurysm • Marginal artery occlusion(2 months later)	SMA stent insertion
2	56/M	Abdominal pain	25	HTN, Hyperlipidemia, A.fib	SMA dissection	• Right renal artery dissection • Left renal artery dissection with ectasia (1 week later)	-
3	70/F	Abdominal pain	15	Hyperlipidemia	Celiac axis and CHA dissection	Stable	-
4	84/F	Abdominal pain	19	HTN,	Aneurysm of PHA, RHA, LHA, LGA, and RGEA right renal artery stenosis	Stable	Right renal artery stent insertion
				A.fib,			
				CHF,			
5	66/M	Abdominal pain	57	None	SMA dissection	• Progression of SMA dissection • Pancreaticoduodenal artery aneurysm (9 days later)	SMA Stent insertion
6	70/F	Abdominal pain	38	None	SMA dissection, Right colic artery dissection, Middle colic artery occlusion	Ileocolic artery aneurysm (1 month later)	-
7	40/M	Lt. flank pain	15	None	Celia axis stenosis, CHA stenosis, Splenic artery occlusion	Stable	-
8	53/F	Abdominal pain	22	None	GDA pseudoaneurysm, Celiac axis dissection	Stable	Embolization

*No*., number; *CT*, computed tomography; *SMA*, Superior mesenteric artery; *CHA*, common hepatic artery; *PHA*, proper hepatic artery; *RHA*, right hepatic artery; *LHA*, left hepatic artery; *LGA*, left gastric artery; *RGEA*, right gastroepiploic artery; *GDA*, gastroduodenal artery; HTN, hypertension; A.fib, atrial fibrillation; CHF, congestive heart failure.

Markers of inflammatory vascular disease, such as anti-neutrophil cytoplasmic antibody (ANCA), complement C3/C4, fluorescent antinuclear antibody (FANA), rheumatoid factor (RF), anti-La antibodies, and anti-cardiolipin antibodies, were all negative. C-reactive protein was elevated in one patient showing a splenic infarct (patient No.7).

### Arterial involvement of SAM

All eight patients were subjected to CT angiography at presentation, identifying 19 lesions as follows: celiac axis, three (37.5%); superior mesenteric artery (SMA), four (50%); common hepatic artery, two (25%); and one each involving right renal, splenic, right colic, middle colic, gastroduodenal, left gastric, right gastroepiploic, proper hepatic, right hepatic, and left hepatic arteries. A number of dissections (n = 8), aneurysms (n = 5), and stenoses or occlusions (n = 5), as well as a single pseudoaneurysm were demonstrated (Table 2).

During follow-up, all patients underwent CT angiography periodically in accord with study protocol. Upon conclusion, 25 lesions ultimately accrued, including three additional aneurysms (SMA branch, pancreaticoduodenal and ilieocolic arteries), marginal artery occlusion, and bilateral renal arterial dissections (Table 2).

### Surveillance results

**Fig 1:**
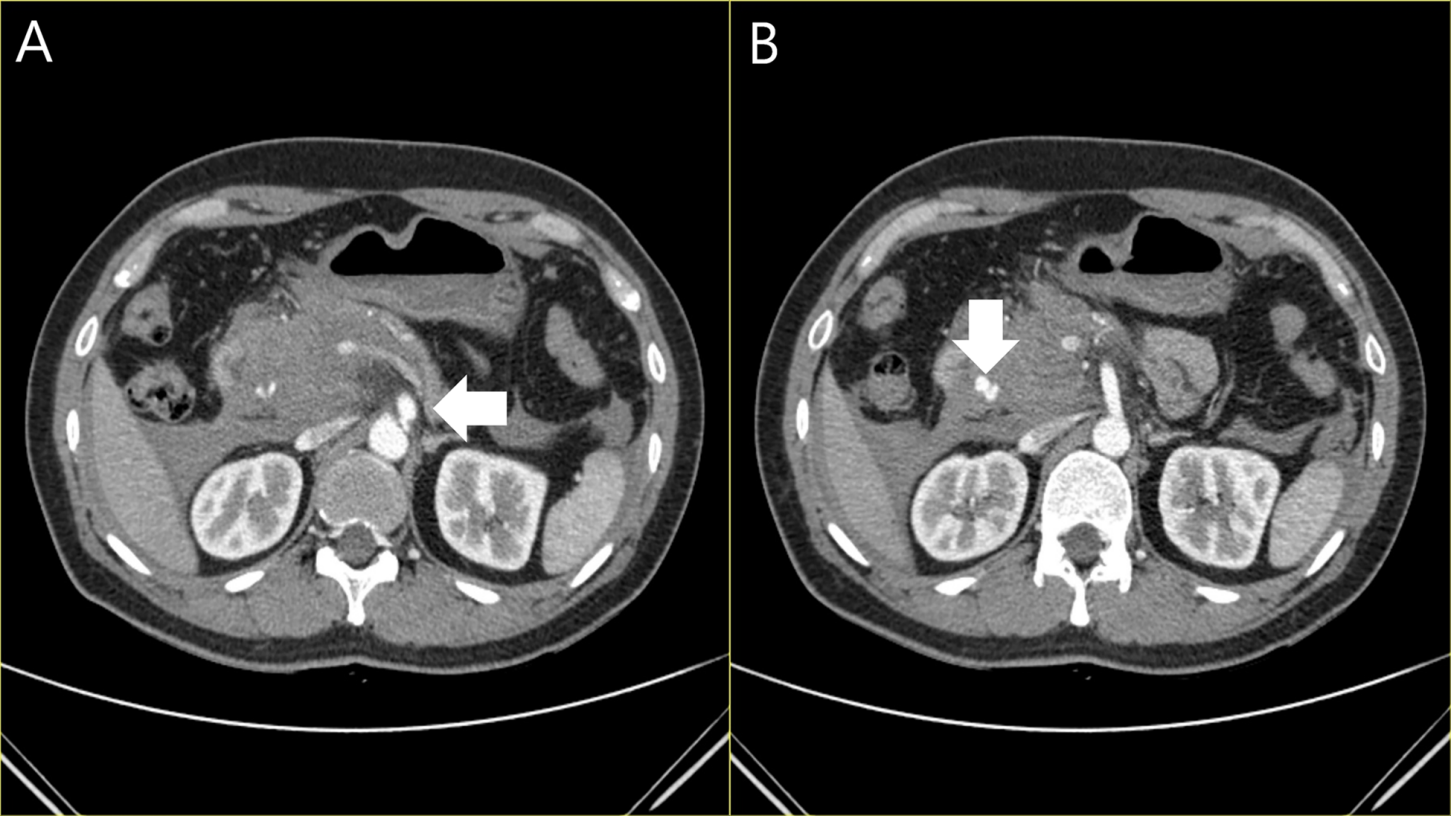
**Initial CT scans (patient No. 8):** (**A**) celiac axis arterial dissection and (**B**) pseudoaneurysm of gastroduodenal artery.

**Fig 2:**
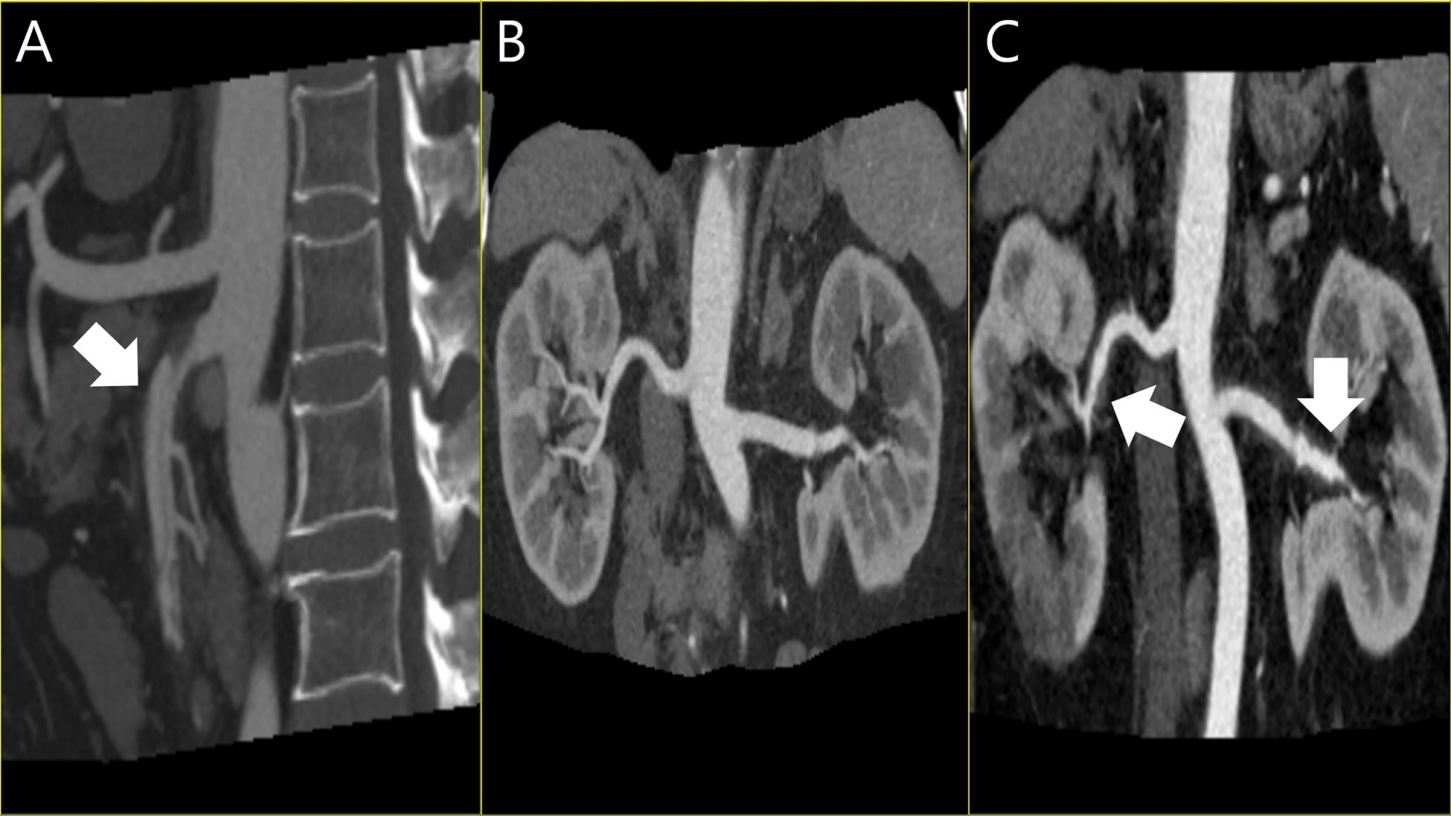
**CT angiography (patient No. 2):** (**A & B**) SMA dissection seen initially, with normal renal vasculature interpreted as symptomatic isolated dissection of superior mesenteric artery until follow-up imaging (1 week later) confirmed (**C**) new dissections of right and left right renal arteries with ectasia.

**Fig 3:**
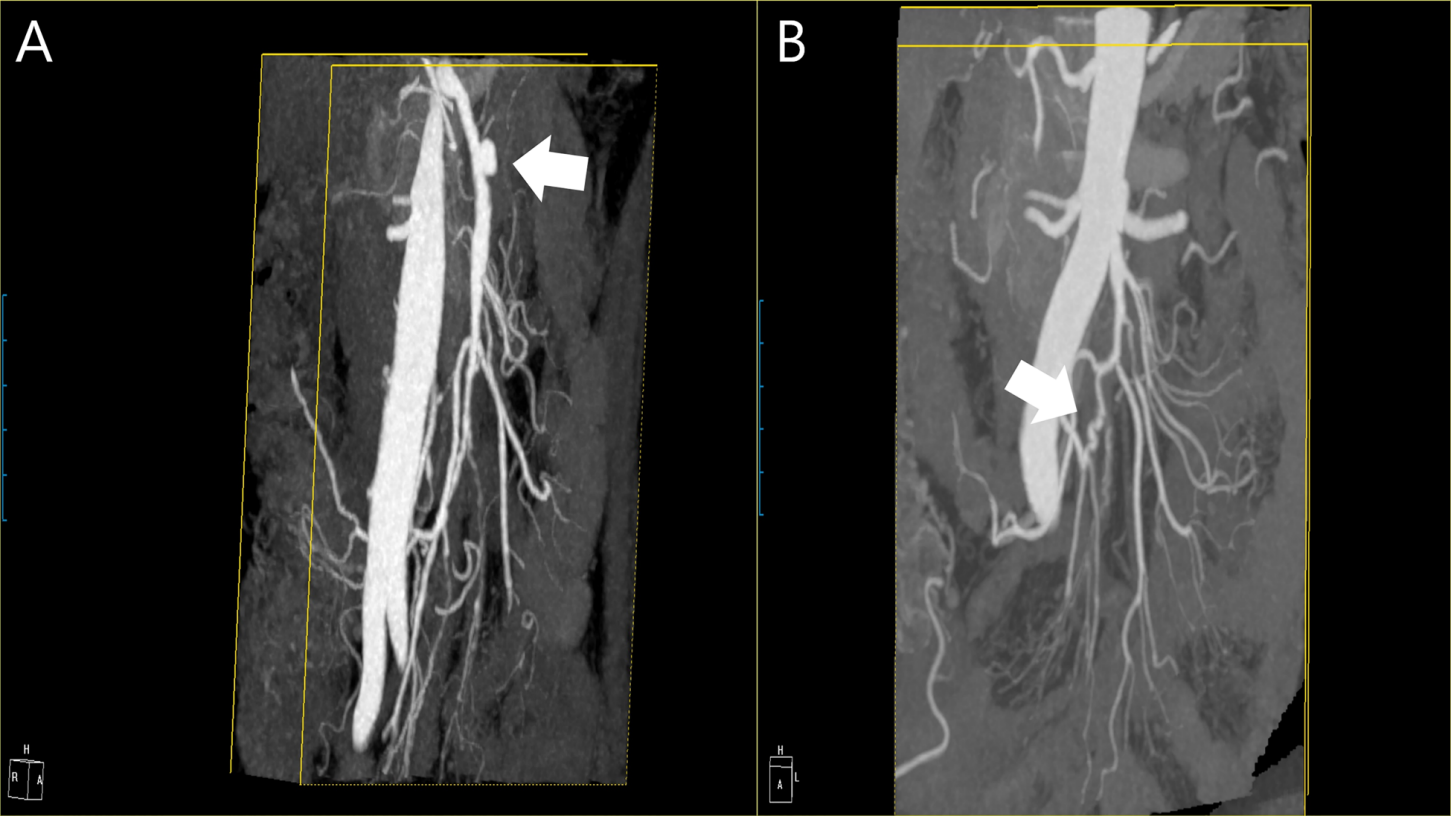
**CT angiography (patient No. 6):** (**A**) SMA dissection at presentation (in addition to right colic arterial dissection and occlusion of middle colic artery); (**B**) newly developed aneurysm of ileocolic artery seen 1 month after initial visit.

Although patient No. 8 underwent endovascular embolization of an initially identified gastroduodenal pseudoaneurysm (Fig 1), other patients opted for regular monitoring. At completion of the follow-up period, lesions in four patients were stable, whereas new arterial lesions developed in the other four (No. 1, 2, 5, 6). In one patient (No. 1), a SMA branch aneurysm and a marginal arterial occlusion appeared at Month 2. Another patient (No. 2), presenting with SMA dissection, displayed dissections of right and left renal arteries with ectasia at Week 1 (Fig 2). Patient No. 5, also presented with SMA dissection and developed a pancreaticoduodenal aneurysm by Day 9. Finally, patient No. 6 developed an ileocolic aneurysm at Month 1 (Fig 3).

Among these patients, symptomatic isolated dissection of SMA (SIDSMA) was first considered in three (No. 1, 2, 5). However, a diagnosis of SAM was made after other splanchnic arterial lesions materialized. Two patients (No. 1, 5) required stent placement due to progression of dissection, as specified in our previously published recommendations [7].

### Literature cases

To date there are 76 studies and 101 cases of SAM (Tables 3 and 4), of which 60 were men and 41 were women. Median age was 56.9 years (range, 0–91 years 95% CI range,). Most patients presented with abdominal pain (68%), whereas eight patients were asymptomatic. In 68 patients (67%), diagnosis was confirmed by histologic means. Others were diagnosed clinically. The vessel most often associated with SAM was splenic artery, accounting for 28% of all SAM involvement. Common hepatic artery (and hepatic branches), celiac trunk, and renal arteries were the next most commonly involved.

**Table 3:** Baseline demographic and clinical characteristics of literature cases.

Variables	Patients (n = 101)	95% CI
Age: median (range), y	56.9 (0–91)	56.9 (53.8–60.0)
Gender (male:female)	60:41	59.4 (49.2–69.1)
Initial presentation as abdominal pain	69 (68%)	68.3 (58.3–77.2)
Histologic diagnosis	68 (67%)	67.3 (57.3–76.3)
Management		
Conservative management	14 (14%)	13.9 (7.8–22.2)
Endovascular intervention	24 (24%)	23.8 (15.9–33.3)
Open surgery after failed endovascular intervention	7 (7%)	6.9 (2.8–13.8)
Open surgery	41 (41%)	40.6 (30.9–50.8)
None mentioned or attempted	15 (15%)	14.9 (8.6–23.3)

**Table 4:** Included studies and outcomes of interest.

Authors	Year	No. of cases	Histologic confirmation	Management	Outcome	SAM involved vessels
Matsuda et al.[8]	2015	1	Yes	O after failed EV	S	Splenic a.
Kimura et al.[9]	2015	1	Yes	O after failed EV	S	Inf. PDA
Liao et al.[10]	2015	1	No	EV	S	SMA
Ruderman et al.[11]	2015	1	No	EV	S	Renal a.
Nishimura et al.[12]	2014	1	No	O	S	Middle colic a.
Galketiya et al.[13]	2014	1	Yes	O	S	Lt. colic a.
Horsley et al.[14]	2014	1	No	C	S	SMA, IMA, ileocolic a. Hepatic a.
Yamamoto et al.[15]	2014	1	No	EV	S	PDA
Marshall et al.[16]	2013	2	1. Yes 2. Yes	1. O after failed EV 2. O after failed EV	1. S 2. S	1. Hepatic a. 2. Hepatic a.
Kidogawa et al.[17]	2013	1	No	C	S	Inf. PDA
Alturkustani et al.[18]	2013	2	1. Yes 2. Yes	1. N 2. N	1. DS 2. DS	1. VA 2. VA
Tabassum et al.[19]	2013	1	Yes	O	S	LGA
Yoshida et al.[20]	2013	1	Yes	O	S	Lt. colic a.
Ushijima et al.[21]	2013	1	No	O	S	Post. inf. PDA
Yoo et al.[22]	2012	1	No	EV	S	SMA, middle colic a.
Matsuda et al.[23]	2012	1	No	O	S	Ant. cerebral a., Lt. VA
Ito et al.[24]	2012	1	No	C	S	SMA, IMA, Lt. renal a. Splenic a.
Hatogai et al.[25]	2012	1	Yes	O	S	Hepatic a., Celiac artery
Cooke et al.[26]	2012	1	No	EV	S	VA
Filippone et al.[27]	2011	2	1. No 2. Yes	1. C 2. O	1. S 2. S	1. Rt. renal a., Rt. carotid a., Both VA, Lt. middle cerebral a. Both renal a. 2. Hepatic a., SMA
Gahide et al.[28]	2011	1	No	EV	S	Lt. renal a.
Obara et al.[29]	2011	1	No	EV	S	Splenic artery, Celiac artery
Oki et al.[30]	2011	1	Yes	O	S	Rt. renal a.
Tomonaga et al.[31]	2011	1	No	C	S	Hepatic a.
Tameo et al.[32]	2011	1	Yes	O after failed EV	S	SMA
Fujiwara et al.[33]	2011	1	Yes	O after failed EV	S	Middle colic a.
Naitoh et al.[34]	2010	1	No	EV	S	Splenic a. Celiac a.
Baker-LePain et al.[35]	2010	2	1. Yes 2. Yes	1. O 2. O	1. S 2. S	1. Hepatic a., Lt. colic a., Splenic a. 2. Celiac a., Splenic a. Hepatic a.
Davran et al.[36]	2010	3	1. No 2. No 3. No	1. EV 2. EV 3. EV	1. S 2. S 3. S	1. Rt. renal a., SMA, Celiac a. 2. Hepatic a., Renal a., SMA 3. SMA, PDA
Soga et al.[37]	2009	1	Yes	C	DN	Renal a.
Ro et al.[38]	2009	1	Yes	N	DS	RGEA, LGA, VA
Keuleers et al.[39]	2009	1	Yes	O	S	Ascending aorta
Kahn et al.[40, 41]	2009	1	Yes	O	DS	Splenic a., Celiac a., Middle colic a.
Agarwal et al.[42]	2009	2	1. No 2. No	1. C 2. EV	1. S 2. S	1. Splenic a., Celiac a. 2. SMA, Renal a., Celiac a., Splenic a., GEA
Hirokawa et al.[43]	2009	1	No	EV	S	Middle colic a.
Ro et al.[44]	2008	1	Yes	N	DS	Vertebral a., Basilar a., Internal carotid a.
Hashimoto et al.[45]	2008	1	Yes	EV (then elective O)	S	Splenic a., GEA, SMA
Abdelrazeq et al.[46]	2008	1	Yes	O after failed EV	S	Marginal a. of Drummond
Shimohira et al.[47]	2008	4	1. No 2. No 3. No 4. No	1. EV 2. EV 3. EV 4. EV	1. S 2. S 3. S 4. S	1. Hepatic a., Splenic a. 2. GEA 3. Middle colic a., Hepatic a., Celiac a. 4. IMA, Lt. colic a.
Mizutani et al.[48]	2008	1	No	EV	S	Rt. renal a.
Takahashi et al.[49]	2007	1	No	EV	S	Middle colic a.
Muller and Kullmann[50]	2006	1	Yes	O	S	Pulmonary arterioles
Rosenfelder et al.[51]	2006	1	Yes	O	S	Colic a., Mid-jejunal a., Hepatic a., GA
Michael et al.[52]	2006	5	1. No 2. No 3. Yes 4. Yes 5. No	1. C 2. EV 3. O 4. N 5. C	1. S 2. S 3. S 4. DS 5. S	1. Celiac a., SMA, Hepatic a. 2. Celiac a., Hepatic a., GDA, Lt. renal a. 3. SMA, GDA, Middle colic a. 4. Middle colic a. 5. SMA, Renal a., Jejunal a.
Phillips and Lepor[53]	2006	1	Yes	O	S	Lt. renal a.
Obara et al.[54]	2006	1	Yes	O	S	Lt. ICA, Celiac a., SMA, Hepatic a.
Imai et al.[55]	2005	1	Yes	N	DS	Splenic a.
Yamakawa et al.[56]	2005	2	1. Yes 2. Yes	1. O 2. O	1. S 2. DS	1. Post. inf. cerebellar a. 2. Post. inf. cerebellar a.
Jibiki et al.[57]	2005	1	Yes	O	S	PDA, Celiac a., Splenic a.
Basso et al.[58]	2005	2	1. Yes 2. Yes	1. O 2. C	1. DS 2. DS	1. Submucosal and serosal intestinal a. 2. Both ICA
Hirakawa et al.[59]	2005	4	1. Yes 2. Yes 3. Yes 4. Yes	1. O 2. O 3. O 4. O	1. U 2. U 3. U 4. U	1. Celiac a., LGA, Splenic a., CHA 2. Celiac a., LGA, Splenic a. 3. Celiac a., LGA 4. Splenic a.
Chino et al.[60]	2004	1	Yes	O	S	Middle colic a.
Nishiyama et al.[61]	2004	1	Yes	N	DS	LGA
Soulen et al.[62]	2004	1	No	EV	S	CHA, Splenic a., Both renal a., GDA, SMA
Rengstorff et al.[63]	2004	1	Yes	O	S	IMA
Yamada et al.[64]	2004	1	Yes	O	S	Lt. common iliac a.
Eifinger et al.[65]	2004	1	Yes	C	DS	Placental a., Umbilical cord a.
Takagi et al.[66]	2003	1	Yes	O	S	Celiac a., Splenic a., Lt. renal a.
Ohta et al.[67]	2003	1	Yes	EV (then elective O)	S	Vertebro-basilar junction, Ant. Communicating a., Lt. superficial temporal a.
Sakata et al.[68]	2002	1	Yes	C	DS	Rt.VA, Lt. ICA, SMA, Bilateral renal a., Lt. EIA
Ryan et al.[69]	2000	1	No	EV	S	Hepatic a.
Chan et al.[70]	1998	1	Yes	O	S	Hepatic a., Splenic a.
Nagashima et al.[71]	1998	1	Yes	N	DS	PHA
Sakano et al.[72]	1997	1	Yes	O	S	Middle colic a.
Kato et al.[73]	1996	1	Yes	O	S	IMA
Peters et al.[74]	1995	1	Yes	C	DS	ICA
Ito et al.[75]	1995	1	Yes	C	DS	Splenic a.
Slavin et al.[1]	1995	5	1. Yes 2. Yes 3. Yes 4. Yes 5. Yes	1. O 2. N 3. O 4. N 5. N	1. S 2. DN 3. S 4. DN 5. DS	1. Lt. colic a. 2. Epicardial coronary a. 3. Ileocolic a. 4. Hepatic a. 5. GEA
Wang and Huang[76]	1994	1	Yes	O	S	Lt. colic a.
Juvonen et al.[77]	1994	1	Yes,	O	S	Omental a., Splenic a., Pancreatic a.
Eskenasy-Cottier et al.[78]	1994	1	Yes	N	DS	Ant. Circulation of the circle of Willis
Inayama et al.[79]	1992	1	Yes	O	S	LGA, Splenic a.
Armas and Donovan[80]	1992	1	Yes	N	DS	Hepatic a.
Heritz et al.[81]	1990	1	Yes	O	S	Omental a., Ileal a., GDA, Renal a.
Slavin et al.[82]	1989	1	Yes	O	S	Jejunal a.
Slavin et al.[4]	1976	3	1. Yes 2. Yes 3. Yes	1. N 2. N 3. N	1. DS 2. DS 3. DS	1. Splenic a. 2. Rt. colic a. 3. Lt. colic a.

*C*, Conservative management; *DN*, died from causes not directly related to segmental arterial mediolysis; *DS*, died as a direct consequence of segmental arterial mediolysis or segmental arterial mediolysis-related sequelae; *EV*, endovascular intervention; *N*, none mentioned or attempted; *O*, open surgery; *S*, survived; *U*, outcome not reported; *No*., number; *SMA*, Superior mesenteric artery; *CHA*, common hepatic artery; *PHA*, proper hepatic artery; *RHA*, right hepatic artery; *LHA*, left hepatic artery; *LGA*, left gastric artery; *RGEA*, right gastroepiploic artery; *GEA*, gastroepiploic artery; *GDA*, gastroduodenal artery; *PDA*, pancreaticoduodenal artery; *GA*, gastric artery; *ICA*, internal carotid artery; *VA*, vertebral artery; *EIA*, external iliac artery.

Of the 94 cases with reported survival outcomes, SAM-related mortality was 22% (21 patients), with 78% (73 patients) surviving acute presentations of SAM. Of the 21 patients who died of SAM-related causes, 13 reportedly died before any intervention could be attempted (i.e., death on arrival to hospital) or without intervention, and five died despite attempts at nonoperative conservative management.

Eventually only 14 patients were conservatively managed. Another 47 patients underwent surgical ligation of bleeding vessels/or resection of aneurysmal segment, often with vascular reconstruction either as primary management or after failed endovascular intervention. Twenty-four patients were managed by endovascular intervention through sole use of coil embolization.

In most cases reviewed, follow-up indicated that patients were largely asymptomatic clinically, with additional imaging showing variable outcomes from complete resolution to no change in untreated aneurysm [5]. Few reports of new arterial lesions appears in the literature.

## Discussion

Slavin et al (1976) were the first to define SAM, describing this pathologically distinct entity based on three autopsies. Fundamentally, SAM involves lytic degeneration of medial smooth muscle leading to tearing/separation from adventitia and adjacent fibrosis. Hence, patients commonly present with aneurysms, dissections, stenoses and ruptures, often requiring emergency surgical or endovascular intervention [32].

The 101 cases of SAM that have been reported thus far likely represent a gross underestimate of its true incidence. Typically, splanchnic vessels of middle-aged and elderly adults are affected in SAM, although carotid, renal, intracranial, and iliac arterial involvement has been reported, and some cases have developed in young individuals. To date, some authorities insist that SAM represents a variant or a precursor of FMD [1, 2]. However, young females are usually afflicted with FMD, showing diffuse disarray of media in mid- and distal arterial segments where smooth muscle is replaced by collagen [6, 83]. Arterial stenoses are common in FMD, but aneurysms and dissections are rare [35, 83]. The differential diagnosis also includes Marfan’s syndrome, polyarteritis nodosa, Takayasu’s arteritis, Behçet’s disease, allergic granulomatous angiitis, and various disorders of collagen (eg, Ehlers-Danlos syndrome, Loeys-Dietz syndrome, and neurofibromatosis) [35]. SAM differs significantly in terms of pathologic findings, laboratory abnormalities, and sites of involvement, predominantly involving splanchnic vasculature and presenting distinctively. Marfan’s and Ehlers-Danlos syndromes commonly manifest as dissections and aneurysms. Loeys-Dietz syndrome also is usually marked by aneurysms. Although neurofibromatosis may produce long-segment stenosis and aneurysms, dissections are rare [35].

In a literature review, abdominal pain was the most common presenting symptom (68%), followed by hemodynamic shock (25%), neurologic symptom (12%), and 11 patients (11%) died before further investigation and/or attempted management. However, eight cases (8%) reported an asymptomatic presentation. In our review, seven of our patients (87.5%) presented with acute abdominal pain and one patient experienced acute flank pain.

Mean age at presentation was slightly higher in our patients, compared with the pooled data of literature review (62.8 vs 56.9 years). Multiple vessels were initially involved in five of our patients (62.5%), as opposed to 47% in other reports [54, 84]. Some sources further suggest that multiple splanchnic vessels are affected during the course of SAM [32]. In support of this tenet, half of our patients developed new lesions of splanchnic arteries (Pt. No. 1, 2, 5, 6) (Table 1). Consequently, careful clinical follow-up is recommended, checking regularly for new arterial lesions via CT angiography. According to our protocol, CT angiography is done at presentation and repeated periodically (Week 1 and Month 3 after diagnosis, then yearly). Only if initial lesions resolve completely within the first year of follow-up is yearly imaging abandoned. In reviewing the literature, only in a few instances did new lesions develop during the course of SAM. A high index of suspicion and careful evaluation of imaging studies are essential in this regard. Our imaging protocol during follow-up periods may certainly played a role here.

Three of our patients (37.5%) were initially considered as SIDSMA in our series. Once new splanchnic arterial lesions materialized, a diagnosis of SAM was eventually established. It may well be that SIDSMA is a preliminary manifestation of SAM. Regular imaging and careful monitoring are thus prudent in instances of SIDSMA as well.

No formal guidelines for management of SAM exist as yet. Patients with shock and intra-abdominal hemorrhage should be treated with emergency surgical or endovascular intervention. If a lesion progresses, increasing the risk of organ ischemia, endovascular intervention is perhaps the foremost therapeutic option [32]. Previous studies have indicated that a benign course of the disease requires no therapy [52]. Given that the arterial walls are already prone to dissection or development of aneurysms, intra-arterial catheter manipulation and balloon dilatation stand to worsen or instigate arterial dissections. Accordingly, invasive procedures may be reserved for patients with hemodynamically unstable conditions or significant end-organ ischemia [47, 63, 69, 85]. Four of our patients submitted to endovascular interventions. Two had stents placed in SMA, adhering to related treatment guidelines of our group; [7] one underwent coil embolization for a pseudoaneurysm of gastroduodenal artery with bleeding; and in another we opted for right renal arterial stent insertion due to progressing azotemia. No surgical interventions were done. The utility of corticosteroids in the management of this disease is questionable, given the absence of inflammation in histologic preparations. Active management of hypertension may otherwise be beneficial [62].

The present study has several limitations. As other case series reports, our initiative deals with a small group of patients (N = 8). The latter may seem abundant by comparison, relative to 101 similar case reports in the literature, but small patient samplings provide only limited perspective of this disease entity. In addition, none of the arterial lesions had pathologic confirmation, which would have been prohibitive. Each diagnosis was based solely on clinical and imaging findings, in conjunction with an absence of laboratory abnormalities and exclusion of diagnostic alternatives [6]. We applied diagnostic criteria of Kalva et al for this study. Nevertheless, these criteria are in need of further validation. The present study was also retrospective in nature, relying upon only eight patients. However, in the only prior study of more than eight patients, CT angiographic data were insufficient. By comparison, our efforts have generated a significant body of information on SAM, addressing diagnosis, therapy, and follow-up management. With increasing awareness of SAM, new studies are coming forward. The clear potential for misdiagnosis means that untold sufferers fail to receive proper treatment or due vigilance. A multicenter observation registry may offer better insights into clinical and imaging characteristics of SAM, accruing sufficient case numbers for prospective investigation.

## Supporting Information

S1 PRISMA Checklist PRISMA Checklist. (PDF)

S1 PRISMA Flow Diagram PRISMA Flow Diagram. (PDF)

S1 Table Search strategy. (DOCX)
