# Supplementary material for: Longitudinal Evaluation of Segmental Arterial Mediolysis in Splanchnic Arteries: Case Series and Systematic Review
Source: PLoS One. 2016 Aug 11;11(8):e0161182. doi: 10.1371/journal.pone.0161182 (PMC4981304; doi:10.1371/journal.pone.0161182)
Supplement: S1 PRISMA Flow Diagram — (PDF) [file pone.0161182.s002.pdf]

Flow Diagram

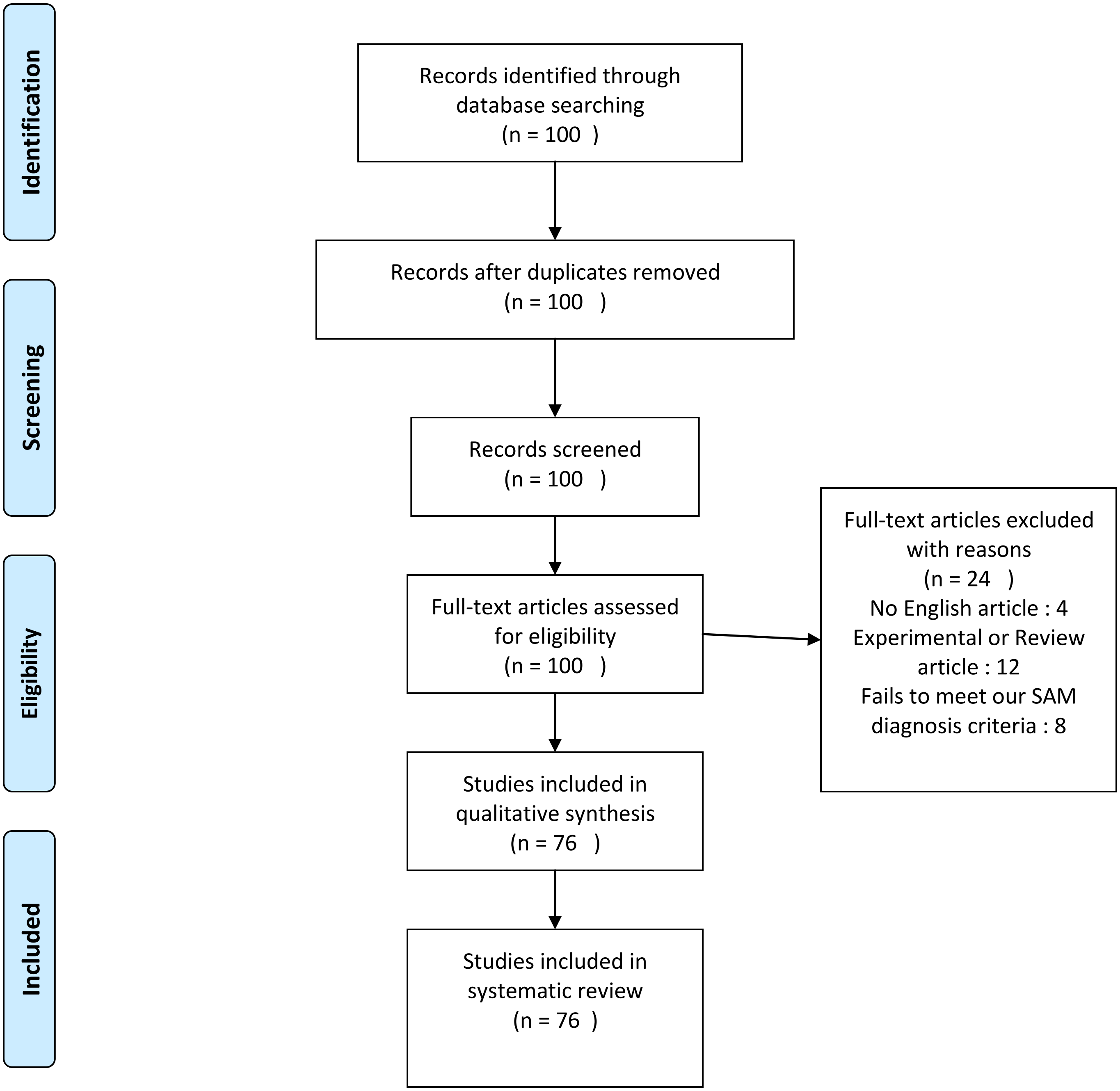

From: Moher D, Liberati A, Tetzlaff J, Altman DG, The PRISMA Group (2009). *P*referred *R*eporting *I*tems for *S*ystematic Reviews and *M*eta-*A*nalyses: The PRISMA Statement. PLoS Med 6(7): e1000097. doi:10.1371/journal.pmed1000097

For more information, visit www.prisma-statement.org.
