# Supplementary material for: Longitudinal Evaluation of Segmental Arterial Mediolysis in Splanchnic Arteries: Case Series and Systematic Review
Source: PLoS One. 2016 Aug 11;11(8):e0161182. doi: 10.1371/journal.pone.0161182 (PMC4981304; doi:10.1371/journal.pone.0161182)
Supplement: S1 Table — (DOCX) [file pone.0161182.s003.docx]

# Search strategy

| Objective | To suggest an optimal management and surveillance strategy in SAM through thorough literature review |
| --- | --- |
| Literature type | Published peer-reviewed articles |
| Study design | All abstracts, case reports, patient series and citations containing clinical presentation, treatment strategy and surveillance protocol of SAM |
| Study settings | Global |
| Key definitions/terms | Segmental arterial mediolysis, segmental mediolytic arteritis |
| Inclusion Criteria | All abstracts, case reports, patient series and citations included if they: (1) reported on at least 1 case of SAM; (2) named vessel(s) involved and characteristic radiologic/histopathologic findings; (3) described any attempted intervention(s) and their outcomes; and (4) reported whether or not the patient survived. |
| Exclusion Criteria | All abstracts, case reports, patient series and citations without definitely diagnosed as SAM pathologically or radiologically |
| Time period | 01-01-1976 to 08-31-2015 |
| Databases | PubMed databases were searched for all articles published in English from 01-01-1976 to 08-31-2015 |
| Languages | English only |
| Search period | Searches will be performed between August and October 2015 |

**Database search terms**

| **Database** | **TEXT / Abst. / keyword** | **Limits/restrictions** |
| --- | --- | --- |
| PubMed | Segmental arterial mediolysis, Segmental mediolytic arteritis | No limitations or restrictions |
